# Supplementary material for: Vectorial Doppler metrology
Source: Nat Commun. 2021 Jul 7;12:4186. doi: 10.1038/s41467-021-24406-z (PMC8263597; doi:10.1038/s41467-021-24406-z)
Supplement: Supplementary file 1 — Supplementary Information [file 41467_2021_24406_MOESM1_ESM.pdf]

Supplementary Information for

Vectorial Doppler Metrology

Liang Fang[+1], Zhenyu Wan[+1], Andrew Forbes[1,2], and Jian Wang[1,*]

[1] *Wuhan National Laboratory for Optoelectronics and School of Optical and Electronic Information, Huazhong University of Science and Technology, Wuhan 430074, Hubei, China.*

[2] *School of Physics, University of the Witwatersrand, Private Bag 3, Johannesburg 2050, South Africa.*

[+] *These author contributed equally to this work.*

* *Corresponding author: jwang@hust.edu.cn*

This PDF file includes:

Supplementary Notes 1-5

Supplementary Figures 1-8

Supplementary Note 1: Classical Doppler effect based on scalar optical fields

In the classical Doppler velocimetry based on scalar optical fields, two light beams as cross-reference are usually used for illumination on a moving object, as shown in Supplementary Fig. 1a. For the convenience of analysis, we assume that the direction of observation is perpendicular to the motion direction. Firstly, we derive the field distribution of interference between two cross-reference light beams along the motion direction of a moving particle. Generally, the complex electric-field function of one light beam projecting in the motion direction can be written by

$$E = A_0(r)\cdot\exp\left[i\left(-\omega t+\mathbf{k}\cdot\mathbf{x}\right)\right], \tag{S1}$$

where $A_0(r)$ is the complex amplitude of electric-field as a function of the spatial position, and ω is the angular frequency of light. $\mathbf{k}$ is the wave vector. For the conventional plane wave, the magnitude of the wave vector is the wavenumber defined as $k=2\pi/\lambda$ with λ being the wavelength, and its direction determines the propagation direction of light or Poynting vector $\mathbf{p}$. Here the particle moves along the $\mathbf{x}$ direction.

As for one of the cross-reference light beams, this projected electric-field in the region of interference can be further written by

$$E_j = A_j(r)\cdot\exp\left[i\left(-\omega t+k\cdot x\cdot\cos\beta_j+\chi_j\right)\right], \tag{S2}$$

where $j=1,\ 2$ represents the beam1 and beam2, respectively. $A_j(r)$ is the real amplitude of electric-field, and χ_j is the initial phase of light beams. x is the position along the motion direction. β_j denotes the angle between the wave vector $\mathbf{k}_j$ and the motion direction $\mathbf{x}$. For simplicity, here we set $\beta_1=\pi/2-\gamma$ for beam1, and $\beta_2=\pi/2+\gamma$ for beam2, where γ is a small angle ($\gamma\approx\sin\gamma$) between the direction of incident light beams and the normal (observation) direction with respect to the trajectory of the moving particle. Thereby, the resulting interference fields between these two cross-reference light beams in Supplementary Fig. 1b can be written by

$$I=\left(E_1+E_2\right)\cdot\left(E_1+E_2\right)^*\approx 2\left|A_0\right|^2\cdot\left[1+\cos 2\left(k\cdot x\cdot\gamma-\alpha\right)\right], \tag{S3}$$

where $\alpha=\left(\chi_2-\chi_1\right)/2$, and the superscript ‘ * ’ stands for complex conjugation. Note that here the complex amplitudes of two cross-reference light beams are approximated as $A_1\approx A_2\approx A_0$. From Eq. (S3), the period of interference fringes can be deduced as $\Lambda=\lambda/2\gamma$ indicated in Supplementary Fig. 1b.

Actually, when a particle moves within one light beam at the velocity of $\mathbf{v}$ along the $\mathbf{x}$, it can scatter light into the detector. Because of normal detection with respect to the motion direction, the Doppler shift of the detected light is just induced by the interaction between the moving particle and the projecting field expressed by Eq. (S1). In

this case, the displacement $\mathbf{x}$ can be replaced by $\mathbf{x}=\mathbf{v}\cdot t$, so from Eq. (S1), the frequency of the scatted light becomes $\omega'=\omega-\mathbf{k}\cdot\mathbf{v}$, and thus the Doppler shift is $\Delta\omega=\omega'-\omega=-\mathbf{k}\cdot\mathbf{v}$. Note that this Doppler shift has a sign of plus or minus corresponding to Doppler blue or red shift, respectively, which is determined by the relative direction of velocity $\mathbf{v}$ with respect to the wave vector $\mathbf{k}$.

Because of the ultra-high angular frequency ω of light, it is difficult to directly detect the shifted frequency of the scattered light (ω'), and the Doppler shift is usually indirectly extracted by the interference with a reference wave (e.g. two cross-reference light beams). Here, the local frequency ω is removed by the beating of two cross-reference light beams, leaving the relatively low frequency Doppler shift. When considering the synchronous interaction of the moving particle with two projecting fields of the cross-reference light beams ($j=1$ and 2) given by Eq. (S2), substituting x with $x=v\cdot t$, where $v=|\mathbf{v}|$, the Doppler signal of the scattered light from interference fields given by Eq. (S3) can be written by

$$I'(v,t)\approx 2|A|^2\left[1+\cos 2(k\gamma vt-\alpha)\right], \tag{S4}$$

where A denotes the complex amplitude of the scattered light into the detector from each of two cross-reference light beams. Thereby, by fast Fourier transform (FFT) for this Doppler signal, the classical linear Doppler shift can be easily extracted as $|\Delta f|=|kv\gamma/\pi|$.

It should be stressed that the case of classical Doppler velocimetry used for detection of the above translational or linear motion can also be extended to the rotational Doppler velocimetry when superposing two beams with orthogonally twisted components $\pm\ell$, where ℓ represents the topological charge number in Supplementary Fig. 1d. In this case, the conventional Poynting vector $\mathbf{p}$ should be modified by adding an azimuthal component, i.e. $\mathbf{p}\to\ell/r\cdot\boldsymbol{\phi}_0+k\cdot\mathbf{z}_0$ in the cylindrical coordinate ($\mathbf{p}_1$ and $\mathbf{p}_2$ for $\pm\ell$), giving the twisted Poynting vector with a skew angle $\gamma=\ell/kr$, with respect to the wave vector $\mathbf{k}$, where $\boldsymbol{\phi}_0$ is the unit vector along the azimuthal direction, $\mathbf{z}_0$ is the unit vector along the wave vector ($\mathbf{k}$) direction, r is the radial position away from the axis. Note that the linear motion can be linked to the rotational motion by a relationship of $v=r\Omega$. According to the Doppler signal given by Eq. (S4), linear Doppler shift is evolved into the rotational Doppler shift, i.e. $|\Delta f|=|kv\gamma/\pi|=|\ell\Omega/\pi|$.

When taking the opposite velocity for the Doppler signal in Eq. (S4), the resulting Doppler signal becomes

$$I'(-v,t)\approx 2|A|^2\left[1+\cos 2(k\gamma vt+\alpha)\right]. \tag{S5}$$

Obviously, the classical Doppler effect based on scalar optical fields shows the symmetry of the Doppler intensity signal, regardless of the phase term α. This makes the directional ambiguity when detecting the moving

object with either linear or rotational motion by the corresponding linear or rotational Doppler effect. Hence, the one-dimensional Doppler signals in Supplementary Fig. 1c and 1e do not carry any direction information of the moving object. Thus, the classical Doppler effect based on scalar optical fields can be regarded as scalar Doppler effect.

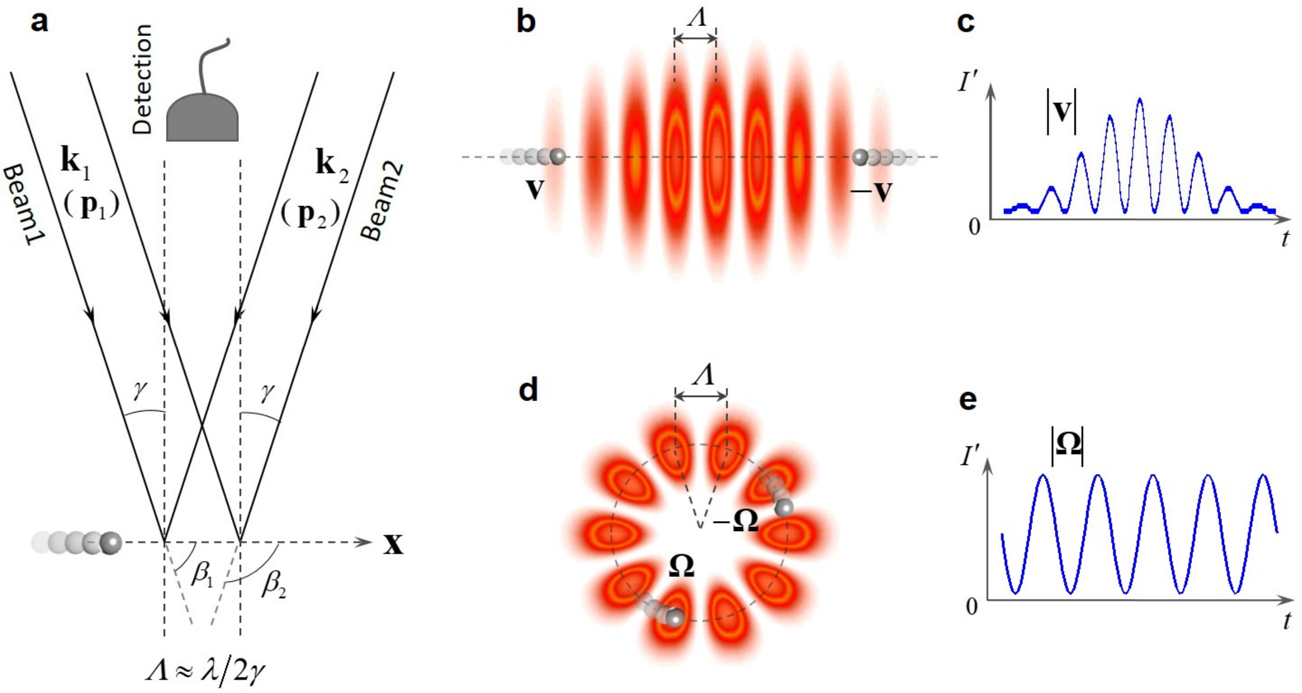

Supplementary Figure 1. Classical Doppler effect based on scalar optical fields in the conventional Doppler velocimetry. a Two cross-reference light beams are used for detection of translational or rotational motion of a moving particle. $\mathbf{k}_1$ and $\mathbf{k}_2$ are wave vectors of two plane-phase beams. $\mathbf{p}_1$ and $\mathbf{p}_2$ are Poynting vectors of two twisted-phase beams. **b** Two plane-phase beams produce straight interference fringes for interaction with translational motion of the particle. **c** Doppler signal of the scattered light by the moving particle with translational motion in interference fields corresponding to (b). **d** Two twisted-phase beams produce rotational interference fringes for interaction with rotational motion of the particle. **e** Doppler signal of the scattered light by the moving particle with rotational motion in interference fields corresponding to (d). Note that the motion direction of the moving particle cannot be distinguished based on the one-dimensional Doppler signals in (c) and (e) that are direction ambiguous.

Supplementary Note 2: Vectorial Doppler effect based on vectorial polarization fields (VPFs)

The classical Doppler effect by scalar optical fields discussed above is not associated with the polarization degree of freedom. In this section, we take consideration of the polarization into the system of Doppler velocimetry. By the same way as the analysis of scalar Doppler effect above, firstly we present how to produce the vectorial polarization fields (VPFs) and then discuss how a moving object interacts with the VPFs, as illustrated in Supplementary Fig. 2a. As for one of two cross-reference light beams with orthogonal circular state of polarization (SoP), the polarized electric-fields of light projecting in the motion direction of the moving object can be expressed using Jones vector as follows

$$\mathbf{E}_j = A_j(r)\cdot\begin{bmatrix}1\\ \sigma_j i\end{bmatrix}\exp\left[i\left(k\cdot x\cdot\cos\beta_j+\chi_j\right)\right], \tag{S6}$$

where $\sigma=+1$ and -1 describes the left-handed and right-handed circular SoP, respectively, and other parameters are the same as those in Eq. (S2).

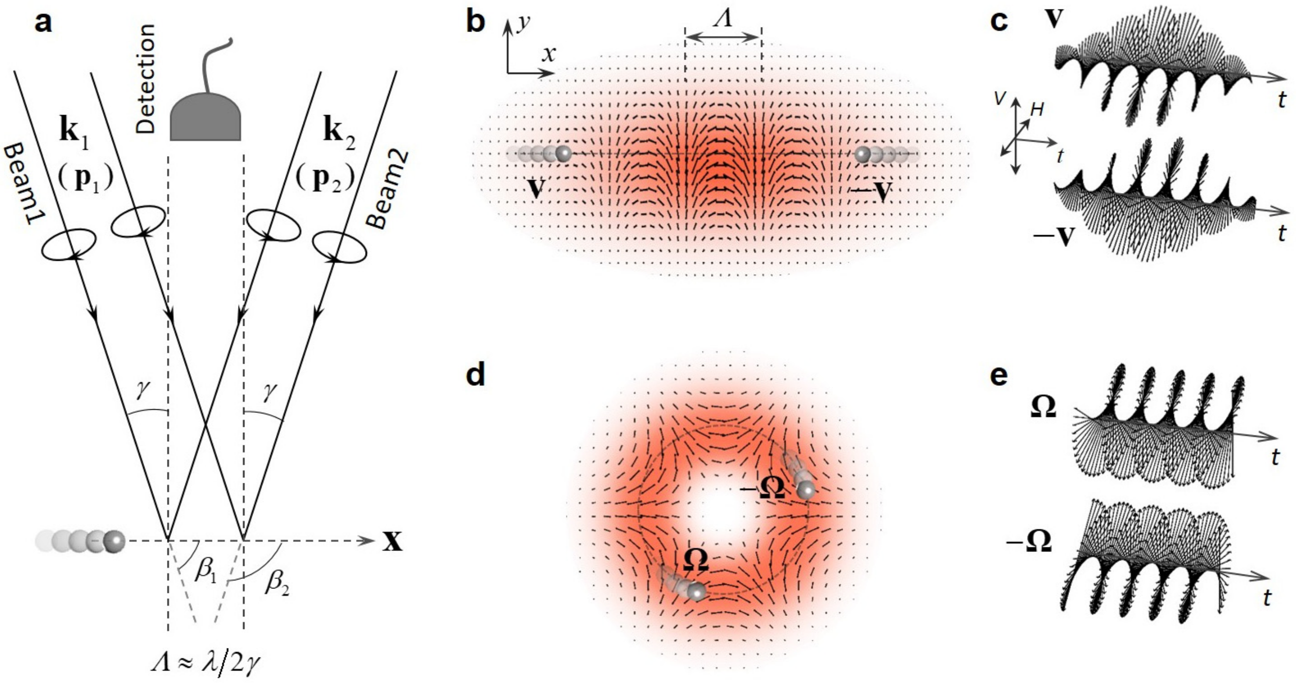

Supplementary Figure 2. Vectorial Doppler effect based on vectorial polarization fields (VPFs). a Two cross-reference light beams with orthogonal circular state of polarization (SoP) are used for detection of velocity vector or pseudovector (magnitude and direction) of a moving isotropic particle. $\mathbf{k}_1$ and $\mathbf{k}_2$ are wave vectors of two plane-phase beams. $\mathbf{p}_1$ and $\mathbf{p}_2$ are Poynting vectors of two twisted-phase beams. **b** Two plane-phase beams superpose into the VPFs along the $\mathbf{x}$ direction for interaction with translational motion vector of the particle. **c** Doppler polarization signals (DPSs) of the scattered light by the moving particle with translational motion vector (opposite directions) in VPFs corresponding to (b). **d** Two twisted-phase beams superpose into the cylindrical VPFs for interaction with rotational motion vector of the particle. **e** DPSs of the scattered light by the moving particle with rotational motion vector (opposite directions) in cylindrical VPFs corresponding to (d). The motion direction of the moving isotropic particle can be clearly distinguished based on the two-dimensional DPSs in (c) and (e) of which the chirality is inversed when reversing the velocity vector.

Along the motion direction of a moving object, these two cross-reference light beams can superpose into the VPFs in Supplementary Fig. 2b as follows

$$\begin{aligned}\mathbf{E}=\mathbf{E}_1+\mathbf{E}_2&=\left\{A_1(r)\cdot\begin{bmatrix}1\\ \sigma_1 i\end{bmatrix}\exp\left[i\left(k\cdot x\cdot\cos\beta_1-\alpha\right)\right]+A_2(r)\cdot\begin{bmatrix}1\\ \sigma_2 i\end{bmatrix}\exp\left[i\left(k\cdot x\cdot\cos\beta_2+\alpha\right)\right]\right\}\exp\left[\frac{1}{2}i\left(\chi_2+\chi_1\right)\right]\\ &\approx A_0(r)\cdot\begin{bmatrix}\cos\left(kx\gamma-\alpha\right)\\ -\sigma\cdot\sin\left(kx\gamma-\alpha\right)\end{bmatrix}\end{aligned}, \tag{S7}$$

where the phase difference $\alpha = (\chi_2 - \chi_1)/2$ determines the initial polarization orientation of VPFs, and the spatial functions with respect to real amplitudes are also approximated as $A_1 \approx A_2 \approx A_0 / 2$, and $\sigma_1 = -\sigma_2 = \sigma$. Note that the phase term of $\exp[i(\chi_2 + \chi_1)/2]$ in Eq. (S7) can be disregarded when deriving the final expression, because it does not affect the polarization distribution of VPFs. Even though the synthesized VPFs (superposed field) shown in Supplementary Fig. 2b may not stably propagate over a long distance in free space because of not satisfying Maxwell's equations, it does not affect the analysis of general VPFs to realize determination of motion vector as a conceptual illustration of vectorial Doppler velocimetry. For these VPFs, the period of spatial polarization variation is also $\Lambda = \pi/(k\gamma) = \lambda/(2\gamma)$. Similar to Supplementary Fig. 1d, for the case of superposition of two twisted-phase light beams, $\gamma = \ell/kr$ and $x = r\phi$, where ϕ is the azimuthal position in the cylindrical coordinate, the general VPFs expressed in Eq. (S7) are simplified to the cylindrical VPFs as

$$\mathbf{E} \approx A_0(r) \cdot \begin{bmatrix} \cos(\ell\phi - \alpha) \\ -\sigma \sin(\ell\phi - \alpha) \end{bmatrix}, \tag{S8}$$

where $\sigma = +1$ describes the cylindrical VPFs analogous to $\mathrm{HE}_{\ell+1,1}$ vector mode, whereas $\sigma = -1$ denotes those analogous to $\mathrm{EH}_{\ell-1,1}$ vector mode, as shown in Supplementary Fig. 2d. Note that it features the spatial polarization orientation inversion between these two kinds of cylindrical VPFs (σ = +1 and -1).

In accordance with Supplementary Note 1, also considering a particle moving at the velocity of $\mathbf{v}$ along the $\mathbf{x}$ within the general VPFs given by Eq. (S7), as shown in Supplementary Fig. 2a. Here isotropic particle is considered. It will scatter the polarized light into the detector. The detected polarized light as two-dimensional Doppler polarization signal (DPS) can be written by

$$\mathbf{E}(v,t) \approx A \cdot \begin{bmatrix} \cos(k\gamma vt - \alpha) \\ -\sigma \cdot \sin(k\gamma vt - \alpha) \end{bmatrix}, \tag{S9}$$

where A is the real amplitude of the detected polarized light. If the moving particle reverses its velocity vector ($\mathbf{v} \to -\mathbf{v}$), the resulting DPS becomes

$$\mathbf{E}(-v,t) \approx A \cdot \begin{bmatrix} \cos(k\gamma vt + \alpha) \\ \sigma \cdot \sin(k\gamma vt + \alpha) \end{bmatrix}. \tag{S10}$$

One can clearly see $\mathbf{E}(-v,t) \neq \mathbf{E}(v,t)$. The DPS shows chirality inversion when reversing the motion direction of the particle within the VPFs, as shown in Supplementary Fig. 2c.

For a particle in rotational motion ($\mathbf{\Omega}$) within the cylindrical VPFs given by Eq. (S8), as shown in Supplementary Fig. 2d, the reflected/scattered DPS can be given by

$$\mathbf{E}(\Omega,t) \approx A \cdot \begin{bmatrix} \cos(\ell\Omega t - \alpha) \\ -\sigma \cdot \sin(\ell\Omega t - \alpha) \end{bmatrix}. \tag{S11}$$

If the moving particle reverses its velocity vector ($\mathbf{\Omega} \to -\mathbf{\Omega}$), the resulting DPS becomes

$$\mathbf{E}(-\Omega,t) \approx A \cdot \begin{bmatrix} \cos(\ell\Omega t + \alpha) \\ \sigma \cdot \sin(\ell\Omega t + \alpha) \end{bmatrix}. \tag{S12}$$

One can also see $\mathbf{E}(-\Omega,t) \neq \mathbf{E}(\Omega,t)$. The DPS also shows chirality inversion when reversing the motion direction of the rotating particle within cylindrical VPFs, as shown in Supplementary Fig. 2e. Apparently, the chiral DPS based on VPFs is a two-dimensional signal, carrying full information (magnitude and direction) of motion vector, belonging to the distinct feature of the vectorial Doppler effect. By using the FFT method and analyzing the relative phase difference (RPD) between the detected intensity signals after two polarizers, the magnitude of the velocity can be deduced and the motion direction can be determined based on the vectorial Doppler effect with spatially variant polarized light fields. By contrast, the direction of the motion vector is not achievable by the one-dimensional Doppler intensity signal from the classical scalar Doppler effect, as shown in Supplementary Fig. 1c and 1e.

Supplementary Note 3: Influence of anisotropic particles on rotation measurement

Remarkably, the above analyses for isotropic particles are based on the assumption that a particle without birefringence does not change the polarization of the reflected/scattered light. This assumption is not rigorous for the more general case of anisotropic particles. As for the interaction between the polarized light and the anisotropic particle, the SoP of the reflected/scattered light from a moving particle is dependent on the anisotropy of the particle such as its shape and birefringence, as well as the spin of the particle around its center of mass in this case. Therefore, the Jones matrix of a spinning anisotropic particle can be given as

$$\begin{aligned} \mathbf{M}(\Theta,t) &= \mathbf{R}(-\Theta t) \cdot \mathbf{M}_0 \cdot \mathbf{R}(\Theta t) \\ &= \begin{bmatrix} \cos\Theta t & -\sin\Theta t \\ \sin\Theta t & \cos\Theta t \end{bmatrix} \begin{bmatrix} m & 0 \\ 0 & n \cdot e^{i\delta} \end{bmatrix} \begin{bmatrix} \cos\Theta t & \sin\Theta t \\ -\sin\Theta t & \cos\Theta t \end{bmatrix}, \end{aligned} \tag{S13}$$

where $\mathbf{R}(\Theta t)$ is the rotation matrix, Θ denotes the spin speed of the anisotropic particle, m and n are the real scatter/reflection coefficients along the short (fast) and long (slow) axes of the anisotropic particle, respectively, as shown in Supplementary Fig. 2a, δ is the optical phase retardation between these two polarization components.

When considering a moving anisotropic particle in both rotational motion and spinning motion (rotational velocity: Ω, spin speed: Θ) within the cylindrical VPFs in Eq. (S8), as shown in Supplementary Fig. 3, the reflected/scattered light by the moving particle features the time-varying SoP (polarization oscillation). It can be understood that the moving particle modulates the polarization of the reflected/scattered light, which can be interpreted as DPS expressed as

$$\begin{aligned}\mathbf{E}(\Omega,\Theta,t) &\approx A\cdot\mathbf{M}(\Theta,t)\cdot\begin{bmatrix}\cos(\ell\Omega t-\alpha)\\ -\sigma\cdot\sin(\ell\Omega t-\alpha)\end{bmatrix}\\ &=\frac{nA}{2}\begin{bmatrix}(\tau+e^{i\delta})\cdot\cos(\ell\Omega t-\alpha)+(\tau-e^{i\delta})\cdot\cos[2\Theta t+\sigma(\ell\Omega t-\alpha)]\\ -\sigma(\tau+e^{i\delta})\cdot\sin(\ell\Omega t-\alpha)+(\tau-e^{i\delta})\cdot\sin[2\Theta t+\sigma(\ell\Omega t-\alpha)]\end{bmatrix},\end{aligned} \tag{S14}$$

where $\tau = m/n$, A is the real amplitude of the reflected/scattered light. Especially, when $\tau = 1$ and $\delta = 0$, it is simplified to the case of the isotropic particle. It is worth noting that for an isotropic particle, whether it spins or not, there is no influence on the rotation measurement.

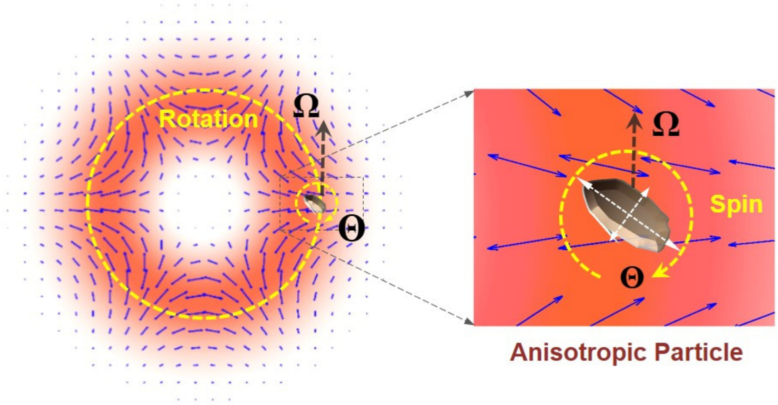

Supplementary Figure 3. Illustration of a moving anisotropic particle in both rotational motion and spinning motion (rotational velocity: Ω, spin speed: Θ) within the cylindrical vectorial polarization fields (VPFs).

Here we focus on the influence of anisotropy (τ and δ) and spin (Θ) of the moving particle on the rotation measurement, as shown in Supplementary Fig. 3. We introduce a method of monitoring the Stokes parameters of DPS reflected/scattered from the cylindrical VPFs for measurement. In general, the Stokes vectors of DPS expressed in Eq. (S14) can be given as

$$S_0 = |E_x|^2 + |E_y|^2, \tag{S15}$$

$$S_1 = |E_x|^2 - |E_y|^2, \tag{S16}$$

$$S_2 = 2|E_x||E_y|\cos\Delta\vartheta, \tag{S17}$$

$$S_3 = 2|E_x||E_y|\sin\Delta\vartheta, \tag{S18}$$

where E_x and E_y are the electric field components polarized along the x and y directions, respectively, given in Eq. (S14), and the phase difference $\Delta\vartheta$ between E_x and E_y is

$$\begin{aligned}\Delta\vartheta &= \tan^{-1}\left[\frac{\mathrm{Im}(E_x)}{\mathrm{Re}(E_x)}\right] - \tan^{-1}\left[\frac{\mathrm{Im}(E_y)}{\mathrm{Re}(E_y)}\right] \\ &= \tan^{-1}\left\{\frac{\sin\delta\cdot[\cos(\ell\Omega t-\alpha)-\cos[2\Theta t+\sigma(\ell\Omega t-\alpha)]]}{(\tau+\cos\delta)\cdot\cos(\ell\Omega t-\alpha)+(\tau-\cos\delta)\cdot\cos[2\Theta t+\sigma(\ell\Omega t-\alpha)]}\right\} \\ &\quad - \tan^{-1}\left\{\frac{\sin\delta\cdot[\sigma\sin(\ell\Omega t-\alpha)-\sin[2\Theta t+\sigma(\ell\Omega t-\alpha)]]}{\sigma(\tau+\cos\delta)\cdot\sin(\ell\Omega t-\alpha)-(\tau-\cos\delta)\cdot\sin[2\Theta t+\sigma(\ell\Omega t-\alpha)]}\right\}\end{aligned}. \tag{S19}$$

When substituting the E_x and E_y components of Eq. (S14) to Eqs. (S15)-(S18), the time-varying Stokes parameters can be simplified as

$$S_0 = \frac{n^2A^2}{2}\left[1+\tau^2+(\tau^2-1)\cos[2\Theta t+2\sigma(\ell\Omega t-\alpha)]\right], \tag{S20}$$

$$S_1 = \frac{n^2A^2}{4}\left\{\begin{aligned}&(1+\tau^2-2\tau\cos\delta)\cdot\cos\left[4\Theta t+2\sigma(\ell\Omega t-\alpha)\right] \\ &+(1+\tau^2+2\tau\cos\delta)\cdot\cos 2(\ell\Omega t-\alpha)+2(\tau^2-1)\cos 2\Theta t\end{aligned}\right\}, \tag{S21}$$

$$S_2 = \frac{n^2A^2}{4}\left\{\begin{aligned}&(\tau^2+1-2\tau\cos\delta)\cdot\sin\left[4\Theta t+2\sigma(\ell\Omega t-\alpha)\right] \\ &-\sigma(1+\tau^2+2\tau\cos\delta)\cdot\sin 2(\ell\Omega t-\alpha)+2(\tau^2-1)\sin 2\Theta t\end{aligned}\right\}, \tag{S22}$$

$$S_3 = n^2A^2\tau\sin\delta\cdot\sin\left[2\Theta t+2\sigma\left(\ell\Omega t-\alpha\right)\right], \tag{S23}$$

where $S_0^2 = S_1^2+S_2^2+S_3^2$. From these equations, one can see that the Stokes parameters (S_1, S_2 and S_3) are the functions of not only the targeted Ω, but also τ, δ and Θ. Especially, for S_1 and S_2, there are three frequency components, $\Delta\omega_1 = 2\ell\Omega$, $\Delta\omega_2 = 2\Theta$ and $\Delta\omega_3 = 4\Theta + 2\sigma\ell\Omega$. Obviously, the three parameters (τ, δ and Θ) related to the anisotropy and spin of the particle would affect the measurement of the rotational velocity. When analyzing the influence of these parameters on the rotation measurement, two cases could be discussed. One is the general case where the anisotropic parameters (τ, δ) of the particle are unknown, and the other is the special case with known anisotropy.

1) For the first case with unknown anisotropy (τ, δ) of the particle, one cannot distinguish the frequency component caused by rotation only ($\Delta\omega_1 = 2\ell\Omega$) among the three Fourier frequencies in S_1 and S_2 ($\Delta\omega_1 = 2\ell\Omega$, $\Delta\omega_2 = 2\Theta$ and $\Delta\omega_3 = 4\Theta + 2\sigma\ell\Omega$). To possibly solve this problem, a feasible method can be considered simply by doubling the topological number ($\ell \to 2\ell$) of the cylindrical VPFs when illuminating the moving particle in the experiment. Because in this case, the frequency component caused by rotation only doubles its value ($\Delta\omega_1 = 2\ell\Omega \to \Delta\omega_1' = 4\ell\Omega$), which can be identified from the three Fourier frequencies ($\Delta\omega_1$, $\Delta\omega_2$ and $\Delta\omega_3$).

Accordingly, the magnitude of the rotational velocity can be deduced from the identified frequency component ($|\Omega| = |\Delta\omega_1 / (2\ell)|$). Meanwhile, the direction of the rotational velocity can be determined through the RPD of the identified frequency component ($\Delta\omega_1$) between S_1 and S_2. Therefore, despite the unknown anisotropy of the particle, one can still extract the contribution of the rotation movement and measure the rotational velocity (magnitude and direction) of the anisotropic particle simply by switching the mode order of the cylindrical VPFs. Similarly, one can also identify the frequency component caused by spin only ($\Delta\omega_2 = 2\Theta$). The magnitude of the spin speed can be deduced from the identified frequency component ($|\Theta| = |\Delta\omega_2| / 2$). The direction of the spin speed can be determined through the RPD of the identified frequency component ($\Delta\omega_2$) between S_1 and S_2.

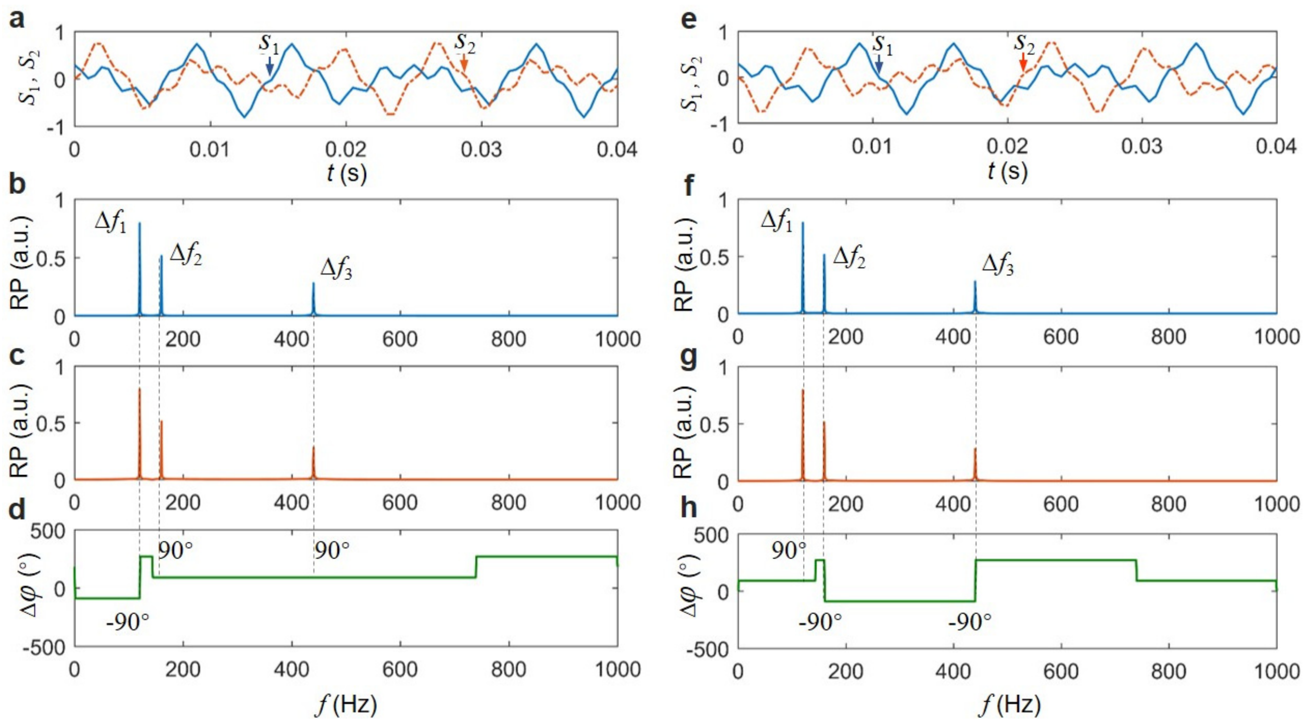

Supplementary Figure 4. Analyses of Stokes parameters (S_1, S_2) for determination of the moving (rotation, spin) particle with known anisotropy (e.g. $\tau = 0.6$, $\delta = \pi/3$) within the HE41-like VPF ($\ell = 3$). a-d Rotational velocity: $\mathbf{\Omega} = -40\pi$ rad/s; spin speed: $\mathbf{\Theta} = -160\pi$ rad/s. **e-h** Rotational velocity: $\mathbf{\Omega} = 40\pi$ rad/s; spin speed of $\mathbf{\Theta} = 160\pi$ rad/s. **a, e** Time-varying Stokes parameters S_1 and S_2. **b, f** The Fourier amplitude-frequency spectra of S_1. **c, g** The Fourier amplitude-frequency spectra of S_2. There are three frequency components ($\Delta f_i = \Delta\omega_i / 2\pi$, $i = 1, 2, 3$), which are distinguishable from their relative amplitudes. Δf_1 and Δf_2 are only rotation- and spin-dependent, respectively. **d, h** The RPD spectra (difference of Fourier phase-frequency spectra between S_1 and S_2). The RPDs of all three frequency components reverse their signs under opposite rotational velocities and spin speeds. RP: relative power; RPD: relative phase difference; a.u.: arbitrary unit.

2) For the second case with known anisotropy (τ, δ) of the particle, one can easily measure the rotational velocity (magnitude and direction) by distinguishing and analyzing the frequency component caused by rotation only

($\Delta\omega_1 = 2\ell\Omega$) in S_1 and S_2. The spin speed (magnitude and direction) can be also measured with distinguished frequency component caused by spin only ($\Delta\omega_2 = 2\Theta$) in S_1 and S_2. Because in this case, different frequency components ($\Delta\omega_1 = 2\ell\Omega$, $\Delta\omega_2 = 2\Theta$ and $\Delta\omega_3 = 4\Theta + 2\sigma\ell\Omega$) in the Fourier frequency spectra of S_1 and S_2 feature different relative amplitudes that can be easily distinguished, as expressed in Eqs. (S21) and (S22) and shown in Supplementary Fig. 4. However, there are some special cases of $\tau = 1$ and $\delta = (2n+1)\pi$ ($n = 0,1,2...$), where the amplitudes of $\Delta\omega_1 = 2\ell\Omega$ and $\Delta\omega_2 = 2\Theta$ become zero and the rotational velocity (Ω) cannot be distinguished from the spin speed (Θ) of the moving particle, because the rotation and spin of the particle give the equivalent contribution to the only one Fourier frequency ($\Delta\omega_3 = 4\Theta + 2\sigma\ell\Omega$) in S_1 and S_2. Nevertheless, this problem could be also solved simply by shifting the laser wavelength in practical measurements so as to avoid these special phase retardations, because the phase retardation (δ) is usually wavelength-dependent for a specific anisotropic particle. If the wavelength-dependent anisotropy is not obvious, we can return to the first case to distinguish and measure the rotational velocity by doubling the mode order of the cylindrical VPFs.

Remarkably, it is a two-step measurement for the first case with unknown anisotropy (τ, δ) of the particle by switching the mode order of the cylindrical VPFs, while a single-step measurement for the second case with known anisotropy (τ, δ) of the particle.

In the practical measurement, the Stokes parameters (S_0, S_1 and S_2) of DPS can be simultaneously obtained by two groups of polarizers and photodetectors, which are used to detect two paths of the divided DPS as follows,

$$S_0 = I_{\mathrm{x}} + I_{\mathrm{y}}, \tag{S24}$$

$$S_1 = I_{\mathrm{x}} - I_{\mathrm{y}}, \tag{S25}$$

$$S_2 = I_{45^\circ} - I_{-45^\circ}, \tag{S26}$$

where I_{x} and I_{y} are the measured light intensity signals by two photodetectors when the first path of DPS passes through two polarizers with their optical axes along the x and y directions, respectively. I_{45° and I_{-45° are the measured light intensity signals by another two photodetectors when the second path of DPS passes through another two polarizers with their optical axes along the 45° and -45° directions, respectively.

Based on the above analyses, one can conclude that it is possible to distinguish the rotational motion and spinning motion of the anisotropic particle and determine the rotational velocity (magnitude and direction) and spin speed (magnitude and direction) by analyzing Stokes parameters, as briefly summarized in Supplementary Tab. 1.

Supplementary Table 1. A summary of determining the rotational velocity and spin speed (magnitude and direction) of a particle based on the time-varying Stokes parameters (S_1 and S_2).

Cases \ Measured values	$\boldsymbol{\Omega}$ (magnitude and direction)	$\boldsymbol{\Theta}$ (magnitude and direction)
First case: τ and δ are unknown	$\ell \to 2\ell$<br>S_1 and S_2: doubled<br>$\Delta\omega_1 = 2\ell\Omega \to 4\ell\Omega$, together with its RPD between S_1 and S_2	$\ell \to 2\ell$<br>S_1 and S_2: fixed $\Delta\omega_2 = 2\Theta$, together with its RPD between S_1 and S_2
Second case: τ and δ are known	S_1 and S_2: distinguishable $\Delta\omega_1 = 2\ell\Omega$ by its amplitude, together with its RPD between S_1 and S_2	S_1 and S_2: distinguishable $\Delta\omega_2 = 2\Theta$ by its amplitude, together with its RPD between S_1 and S_2
Special case: $\tau = 1$, $\delta = (2n+1)\pi$ ($n = 0, 1, 2...$)	Shifting wavelength or returning to the first case	Shifting wavelength or returning to the first case

In addition to the analyses of Stokes parameters, we also study the moving anisotropic particle with only two polarizers and two photodetectors (similar method in the main text). When the DPS ($\mathbf{E}(\Omega,\Theta,t)$) in Eq. (S14) passes through the polarizer with the polarizing angle θ_j (with respect to the x axis), the obtained intensity signal with oscillation can be deduced as

$$\begin{aligned} I_j(\Omega,\Theta,t) &= \left| \begin{pmatrix} 1 & 0 \\ 0 & 0 \end{pmatrix} \cdot \begin{pmatrix} \cos\theta_j & \sin\theta_j \\ -\sin\theta_j & \cos\theta_j \end{pmatrix} \cdot \mathbf{E}(\Omega,\Theta,t) \right|^2 \propto \\ & \frac{n^2A^2}{4}\left(1+\tau^2\right) + \frac{n^2A^2}{8}\left(1+\tau^2+2\tau\cos\delta\right)\cos 2\left(\ell\Omega t + \sigma\theta_j - \alpha\right) \\ & + \frac{n^2A^2}{8}\left(1+\tau^2-2\tau\cos\delta\right)\cos 2\left(\ell\Omega t + 2\sigma\Theta t - \sigma\theta_j - \alpha\right) \\ & + \frac{n^2A^2}{4}\left(1-\tau^2\right)\cos 2\left(\ell\Omega t + \sigma\Theta t - \alpha\right) + \frac{n^2A^2}{4}\left(1-\tau^2\right)\cos 2\left(\Theta t - \theta_j\right) \end{aligned}, \quad \text{(S27)}$$

where j = 1, 2 denotes polarizer 1 and 2, respectively.

The detected intensity signal in Eq. (S27) has four frequency components, i.e. $\Delta\omega_1 = 2\ell\Omega$, $\Delta\omega_2 = 2\Theta$, $\Delta\omega_3 = 4\sigma\Theta + 2\ell\Omega$ and $\Delta\omega_4 = 2\sigma\Theta + 2\ell\Omega$. Note that $\Delta\omega_1$ is caused by rotational motion only, while $\Delta\omega_2$ by spinning motion only. The phases of three frequency components ($\Delta\omega_1$, $\Delta\omega_2$ and $\Delta\omega_3$) are associated with the polarizing angle θ_j. When using two polarizers with a polarizing angle difference $\Delta\theta = \theta_2 - \theta_1 \neq \pm\pi/2$, one can determine the sign of these frequency components ($\Delta\omega_1$, $\Delta\omega_2$ and $\Delta\omega_3$) by analyzing their RPD between the two detected intensity signals ($I_1(\Omega,\Theta,t)$, $I_2(\Omega,\Theta,t)$) after two polarizers, which can be used to further determine the direction of the rotational motion and spinning motion. Following the above similar analyses (Stokes parameters) for the first case with unknown anisotropy (τ, δ) of the particle and second case with known anisotropy (τ, δ) of

the particle, one can also distinguish the rotation and spin of the anisotropic particle and measure the rotational velocity (magnitude and direction) and spin speed (magnitude and direction).

According to the analyses above, it is shown that the moving anisotropic particle with rotational motion and spinning motion can be measured using the method of analyzing Stokes parameters or the method of two polarizers. By comparison, the method of two polarizers is simple with less components (polarizers and photodetectors). The four frequency components may bring a certain degree of disturbance when distinguishing the one caused by rotational motion only and the one by spinning motion only. In a sense, the method of using two polarizers can be regarded as a simplified version of the method of analyzing Stokes parameters. The latter one measuring Stokes parameters gives the complete information of state of polarization (full Poincaré sphere), as clearly listed in Eqs. (S15)-(S18). There are only three frequency components in S_1 and S_2, which make it relatively easier to distinguish the one caused by rotational motion only and the spinning motion only. In addition, for the case with unknown anisotropy of the particle, one might measure the anisotropic parameters (τ , δ) by amplitude ratios of different frequency components in S_0-S_3 based on comprehensive analyses of Stokes parameters in Eqs. (S15)-(S18).

Supplementary Note 4: Experimental setup for the generation of VPFs

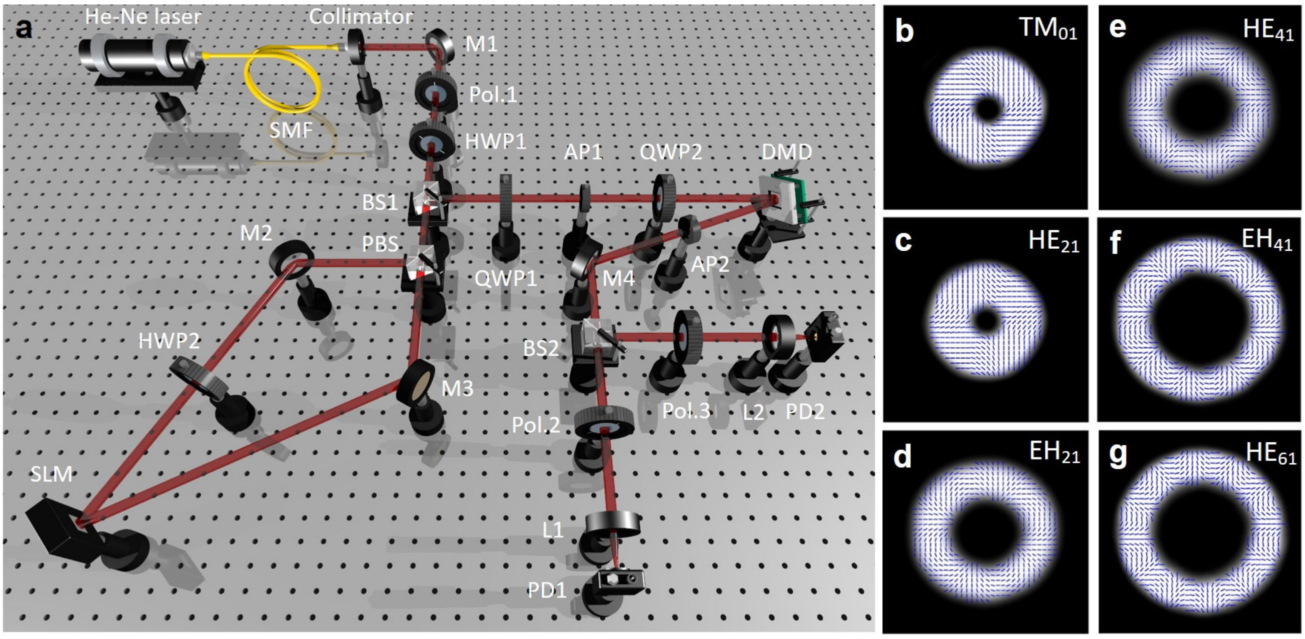

Supplementary Figure 5. Experimental setup and the generation of cylindrical VPFs for the detection of motion vector using vectorial Doppler effect. a Experimental setup. A Sagnac interferometer configuration incorporating an SLM is used to generate VPFs. SMF: single-mode fiber; M: mirror; Pol.: polarizer; HWP: half-wave plate; BS: beam splitter; PBS: polarization beam splitter; SLM: spatial light modulator; QWP: quarter-wave plate; AP: aperture; DMD: digital micromirror device; L: lens; PD: photodetector. **b-g** Measured intensity and polarization distributions of TM_{01}-like (b), HE_{21}-like (c), EH_{21}-like (d), HE_{41}-like (e), EH_{41}-like (f) and HE_{61}-like (g) cylindrical VPFs.

Supplementary Note 5: Additional measured results

In the main text, we present the experimental results for detecting rotational velocities under opposite directions using the cylindrical VPF analogous to HE_{41} vector mode. As well known, this cylindrical VPF consists of light components with the same twisted-phase basis as the EH_{21} vector mode. The cylindrical VPF analogous to EH_{21} vector mode can be generated simply by exchanging the circular SoP between two twisted-phase components used for synthesizing HE_{41}-like VPF. In the experiment, we also employ EH_{21}-like VPF to detect the same angular velocities, as shown in Supplementary Fig. 6. Compared to the measured results using HE_{41}-like VPF in Supplementary Fig. 3d and 3e, the measured two Doppler intensity signals using EH_{21}-like VPF in Supplementary Fig. 6a and 6d just reverse the relative phase difference that is clearly shown in the relative phase spectra in Supplementary Fig. 6c and 6f. The full information (magnitude and direction) detection of motion vector is also achievable using EH_{21}-like VPF.

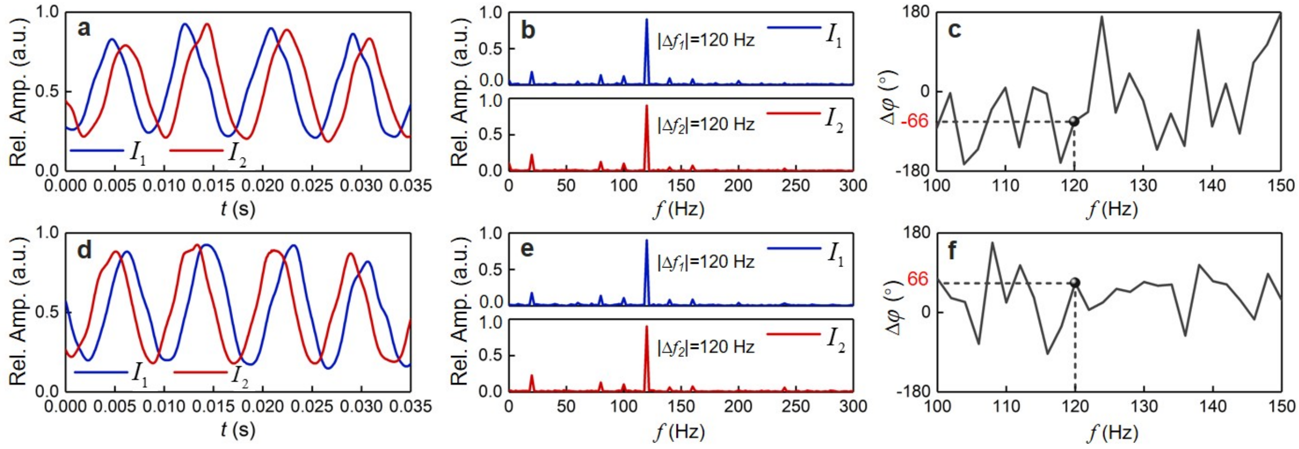

Supplementary Figure 6. Measured results for vectorial Doppler effect based on cylindrical VPFs analogous to EH_{21} vector mode. **a**, **d** Measured Doppler intensity signals by two photodetectors after filtering the DPS through two polarizers. **b**, **c**, **e**, **f** Doppler frequency spectra acquired by fast Fourier transform (FFT) of the recorded Doppler intensity signals in (a) and (d). **b**, **e** Amplitude spectra. **c**, **f** Relative phase spectra showing relative phase difference between two path frequency signals. **a-c** $\mathbf{\Omega} = 40\pi$ rad/s. **d-f** $\mathbf{\Omega} = -40\pi$ rad/s.

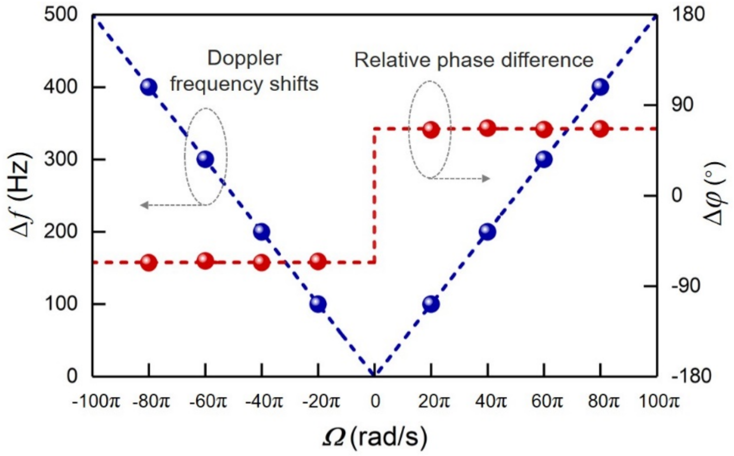

Supplementary Figure 7. Measured results (Doppler frequency shifts and phase shifts) for vectorial Doppler effect based on cylindrically VPFs analogous to HE_{61} vector mode under different rotational velocities. The dots represent measured results and the dashed lines denote theories.

We also experimentally demonstrate the variance of the Doppler shift and the invariance of the relative phase difference under different rotational velocities of the rotating particle using the cylindrical VPF analogous to HE_{61} vector mode, as shown in Supplementary Fig. 7.

It clearly shows that the Doppler shift is proportional to the magnitude of motion velocity, while the relative phase difference depends on the direction of motion vector.

Moreover, we further present the experimental results of real-time angular position and velocity determination of a moving particle in random rotation around an axis using the cylindrical VPF analogous to $HE_{19,1}$ (Supplementary Fig. 8). The intensity spectra with relative phases are measured in Supplementary Fig. 8a. With the knowledge of knowing the starting position and continuous tracking, by counting the peaks and meanwhile estimating the relative phase, according to Eqs. (9) and (10) in the main text, the instantaneous angular positions and rotational velocities can be acquired in Supplementary Fig. 8b and 8c. All experimental results of these additional dates in this section are in good agreement with the theoretical prediction.

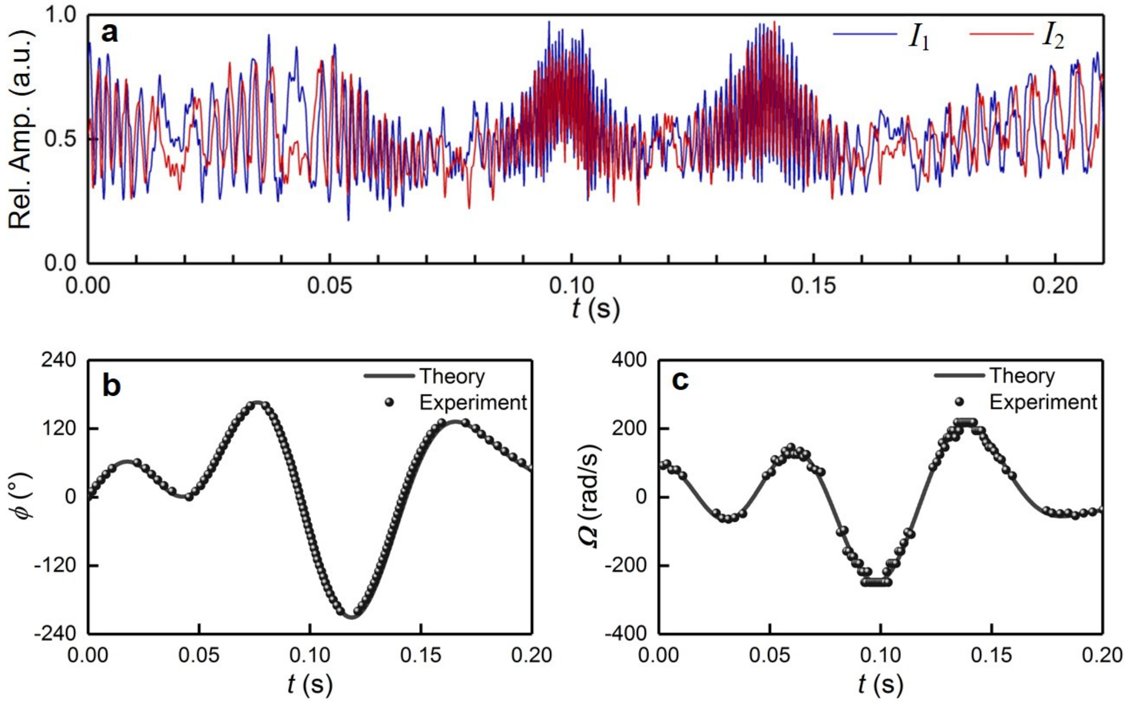

Supplementary Figure 8. Real-time tracking of angular position and rotational velocity for a moving particle in random rotation around an axis using the cylindrical VPF analogous to $HE_{19,1}$, provided that the starting position of the particle is given and the particle is continuously tracked. a The measured intensity signals with relative phases after filtering this DPS through two polarizers. **b** Acquired instantaneous angular positions. **c** Acquired instantaneous rotational velocities. The instantaneous angular positions (b) and rotational velocities (c) are obtained based on the measured intensity signals with relative phases, which are in good agreement with the predicted values.
